# Nature of the Hydrogen Bond Enhanced Halogen Bond

**DOI:** 10.3390/molecules26071885

**Published:** 2021-03-26

**Authors:** Susana Portela, Israel Fernández

**Affiliations:** Departamento de Química Orgánica I and Centro de Innovación en Química Avanzada (ORFEO-CINQA), Facultad de Ciencias Químicas, Universidad Complutense de Madrid, 28040 Madrid, Spain; sportela@ucm.es

**Keywords:** halogen bond, bonding analysis, energy decomposition analysis, density functional theory

## Abstract

The factors responsible for the enhancement of the halogen bond by an adjacent hydrogen bond have been quantitatively explored by means of state-of-the-art computational methods. It is found that the strength of a halogen bond is enhanced by ca. 3 kcal/mol when the halogen donor simultaneously operates as a halogen bond donor and a hydrogen bond acceptor. This enhancement is the result of both stronger electrostatic and orbital interactions between the XB donor and the XB acceptor, which indicates a significant degree of covalency in these halogen bonds. In addition, the halogen bond strength can be easily tuned by modifying the electron density of the aryl group of the XB donor as well as the acidity of the hydrogen atoms responsible for the hydrogen bond.

## 1. Introduction

Noncovalent interactions are ubiquitous in practically all fields of chemistry [1]. In particular, halogen bonding [2,3,4], defined as the interaction between an electrophilic halogen substituent (called “XB donor”) and a nucleophilic Lewis base (called “XB acceptor”) [5], has found applications in different fields such as crystal engineering [6], molecular and anion recognition [7], peptide chemistry [8], and even organocatalysis [9,10]. The so-called σ-hole [11,12] is typically considered as the factor responsible for the ability of halogen atoms to accept electron density from the Lewis base [13]. This σ-hole becomes enhanced (i.e., more positive) as the halogen atom becomes more polarizable and less electronegative. For this reason, the strength of a halogen bond typically follows the order F < Cl < Br < I.

**Figure 1:**
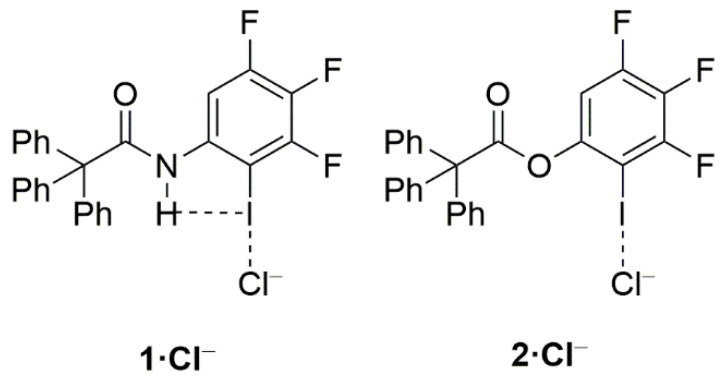
Representative systems exhibiting hydrogen bond-enhanced halogen bonds (HBeXBs) reported by Berryman and co-workers (see reference [14]).

Quite recently, Berryman and co-workers elegantly showed by means of solid-state, solution and computational studies that the strength of the halogen bond can be significantly enhanced when the halogen donor operates simultaneously as a halogen bond donor (C—X⋯Lewis base) and a hydrogen bond acceptor (H⋯X—C) [14]. The term *Hydrogen Bond enhanced Halogen Bond* (HBeXB) was coined to refer to this phenomenon. Typically, it is found that the halogen bond becomes shorter in HBeXB species as compared to their non-hydrogen bond analogues, which is translated into a stronger halogen bond strength. For instance, for the **1·Cl^−^** complex, the instantaneous interaction energy (i.e., the energy difference between the complexes and the isolated constituents in the same geometry as the complex) is ca. 2 kcal/mol higher (more negative) than that computed for its non-hydrogen bond counterpart **2·Cl^−^** (Figure 1). According to the computed electrostatic potential maps, this is mainly ascribed to the more positive σ-hole in **1** as compared to **2**. Similar values were found for related systems where the aryl group in **1·Cl^−^** was replaced by a cationic pyridinium moiety as well as complexes with different halides (F^−^, Cl^−^, Br^−^ or I^−^) as XB acceptors. 

Despite that, very little is known about the nature of this hydrogen bond-enhanced halogen bond phenomenon, i.e., the actual factors responsible for the enhancement of the halogen bond strength, due to the occurrence of an adjacent hydrogen bond. For this reason, herein we decided to apply state-of-the-art computational methods to quantitatively understand the nature of this HBeXB effect.

## 2. Results and Discussion

**Figure 2:**
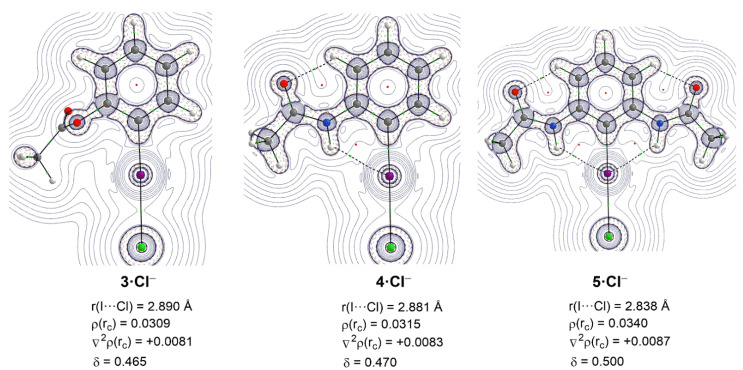
Contour line diagrams ∇^2^ρ(r) for complexes **3·Cl^−^**, **4·Cl^−^**, and **5·Cl^−^** in the C−I–Cl plane. The solid lines connecting the atomic nuclei are the bond paths while the small green and red spheres indicate the corresponding bond critical points and ring critical points, respectively.

First, we compared the chloride complexes involving the parent halogen bond donor **4** and its non-hydrogen bond analogue **3**. Our dispersion corrected B3LYP-D3/def2-TZVPP calculations indicates that the I⋯Cl^−^ distance becomes shorter in the **4·Cl^−^** complex (2.881 Å) than in **3·Cl^−^** (2.890 Å, see Figure 2), which is consistent with the findings by Berryman and co-workers [14]. The halogen bond becomes significantly shorter in the analogous complex **5-Cl^−^** (2.838 Å), which possesses two adjacent hydrogen bonds. As a result, the corresponding Wiberg Bond Indices given by the Natural Bond Orbital (NBO) method [15] increase in the order **3·Cl^−^** < **4·Cl^−^** < **5·Cl^−^** (WBI = 0.230, 0.232 and 0.251, respectively). This simple structural comparison indicates that the strength of the halogen bond increases in the order **3·Cl^−^** < **4·Cl^−^** < **5·Cl^−^**, which, in line with the findings by Berryman and co-workers [14], confirms that the occurrence of an adjacent hydrogen bond enhances the strength of the halogen bond. 

More quantitative insight into the bonding in these species can be gained by means of the QTAIM (Quantum Theory of Atom in Molecules) method [16]. Figure 2 depicts the Laplacian distribution in the C—I—Cl plane for the above complexes. In all cases, the occurrence of I⋯Cl halogen bond is confirmed by the presence of a (3,-1) bond critical point (BCP) between both atoms together with a bond path (BP) running between the XB donor and the XB acceptor. For complexes **4·Cl^−^** and **5·Cl^−^**, there also exist BCPs and BPs between the amide NH group and the iodine XB donor, which confirms the occurrence of the intramolecular NH⋯I hydrogen bonds. 

The data computed using the AIM method clearly supports the relative strength of the halogen bond commented above. Thus, the electron density, ρ(r_c_), and the Laplacian of the electron density, ∇^2^ρ(r_c_), increase in the order **3·Cl^−^** < **4·Cl^−^** < **5·Cl^−^**. Interestingly, the small positive ∇^2^ρ(r_c_) values computed for the BCPs in all species indicate that the charge is locally depleted, a situation which typically corresponds to closed-shell (i.e., donor-acceptor) interactions such as the considered noncovalent interactions. Moreover, the computed delocalization index (δ), which has been suggested as a relative measure of the bond strength [17], also follows the same trend.

To complement the data obtained with the NBO and AIM methods, we next applied the Energy Decomposition Analysis (EDA) method [18,19]. This method has been extensively used to quantitatively understand the nature of the chemical bond in general [18,19], and noncovalent interactions, including halogen bonding, in particular [20,21,22,23]. Table 1 gathers the different EDA-contributors to the total interaction energies between the XB donors **3**, **4**, and **5** and the XB acceptor, Cl^−^. As shown in Table 1, the instantaneous interaction energy (ΔE_int_) in **3·Cl^−^** is 3.1 kcal/mol lower than that in **4·Cl^−^**, which is fully consistent with the data reported by Berryman and co-workers [14]. Interestingly, the computed ΔE_int_ is much stronger in the **5·Cl^−^** complex, featuring two intramolecular NH⋯I hydrogen bonds (ΔΔE_int_ ~ 10 kcal/mol with respect to **3·Cl^−^**). This result suggests that the enhancement of the halogen bond strength by adjacent hydrogen bonds is not additive but occurs in a synergistic manner. 

Moreover, the EDA data indicate that the halogen bond in these complexes is not merely electrostatic but exhibits a high degree of covalency, as can be seen from the high contribution of the ΔE_orb_ term (ranging from 42 to 47% to the total ΔE_int_ term). A similar result was found in other halogen bonds [20,24,25], and in particular, in systems involving carbenes [26] and more recently, also carbones [27]. Interestingly, the electrostatic interactions as well as the orbital interactions (albeit to a lesser extent) steadily increase with the number of adjacent hydrogen bonds in the system (i.e., none < one < two). This is exactly the same trend as that found for the ΔE_int_ term. Therefore, it can be concluded that the enhancement of the halogen bond strength by adjacent hydrogen bonds directly results from stronger electrostatic as well as orbital (although to a lesser extent) attractions between the XB donor and XB acceptor. The contribution of the dispersion forces to the bonding can be considered negligible in all cases (ΔE_disp_ ~ −0.5 kcal/mol).

**Figure 3:**
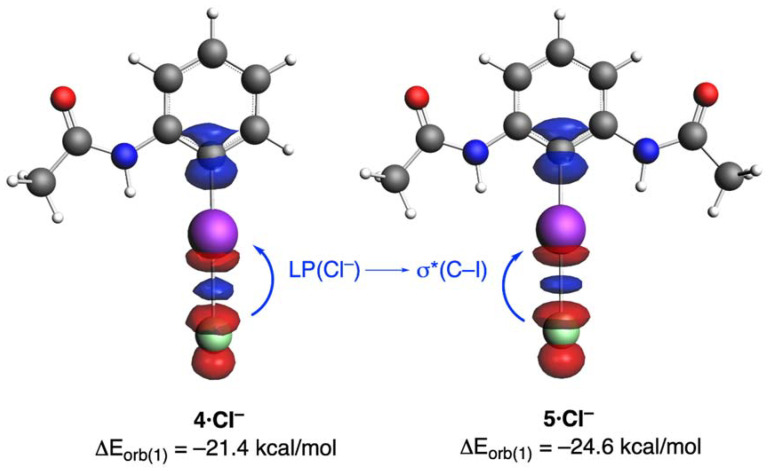
NOCV-deformation densities and associated stabilization energies computed for complexes **4·Cl^−^** and **5·Cl^−^**. The charge flow takes place in the direction red → blue.

We also applied the Natural Orbital for Chemical Valence (NOCV) [28] to identify and quantify the main orbital interactions contributing to the total ΔE_orb_ term. This approach identifies a main orbital interaction (ΔE_orb(1)_) that dominates the total orbital interactions (ca. 70–75%, see Table 1), namely the donation of electron density from the LP(Cl^−^) to the σ*(I—C) molecular orbital (see Figure 3). Not surprisingly, the strength of this molecular interaction follows the same trend as the total orbital interactions, which indicates that the electrophilicity of the XB donor, i.e., its ability to accept electron density from the chloride anion, increases by the occurrence of the adjacent hydrogen bonds: ΔE_orb(1)_ = −20.6 kcal/mol (**3·Cl^−^**) < −21.4 kcal/mol (**4·Cl^−^**) < −24.6 kcal/mol (**5·Cl^−^**). A similar result was found when applying the Second Order Perturbation Theory (SOPT) of the NBO method. Indeed, the corresponding SOPT stabilization energies (ΔE^(2)^) involving the LP(Cl^−^)→σ*(I—C) two-electron delocalization also increases in the order −25.5 kcal/mol (**3·Cl^−^**) < −26.1 kcal/mol (**4·Cl^−^**) < −30.2 kcal/mol (**5·Cl^−^**)

Once the main features of the nature of HBeXB have been disclosed, we next explore the influence of substituents in the aromatic ring on the halogen bond. To this end, we added different electron-donor and electron-withdrawing groups at the *para*-position relative to the iodine atom in the parent XB donor **4** (Table 2). Fore completeness, we also included the experimentally prepared trifluorinated and pyridinium systems **1** and **6** [14].

**Figure 4:**
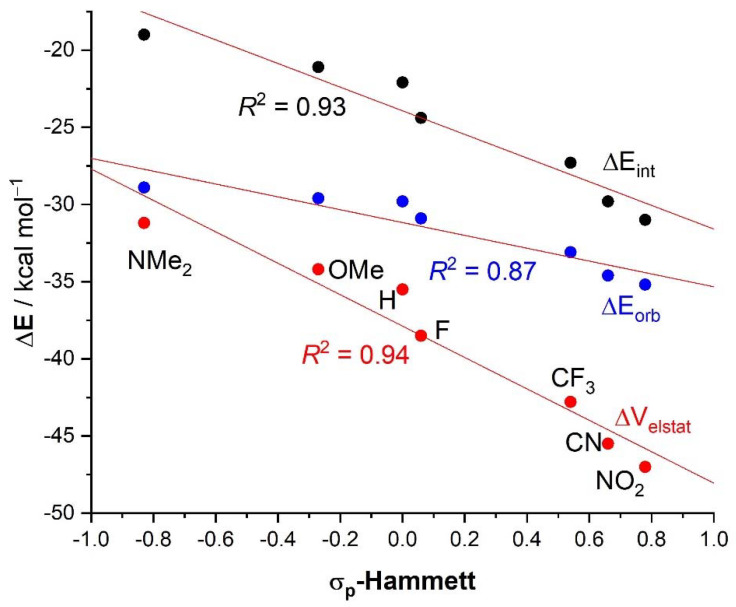
Plot of the energy terms, ΔE_int_ (black circles), ΔV_elstat_ (red circles), and ΔE_orb_ (blue circles) vs the σ_p_-Hammett substituent constants.

In agreement with recent works on the enhancement of the Lewis acidity in halogen donors [29,30], our EDA data confirms that the interaction energy between the XB donor **4** and the chloride anion increases when electron-withdrawing groups are placed in the *para*-position to the iodine atom. Thus, an increase of up to ca. 9 kcal/mol in ΔE_int_ with the respect to the parent **4·Cl^−^** complex (R = H) was computed for the **4-NO_2_·Cl^−^** system, having the strongest electron-withdrawing substituent of the entire series. The opposite is found when placing electron donor groups instead, i.e., the weakest interaction is found for **4-NMe_2_·Cl^−^**, having the good electron-donor NMe_2_ group. This confirms that the strength of the HBeXB can be easily tuned by modifying the electron density of the aryl group. The computed data therefore indicates that the halogen bond strength steadily increases when the electron-withdrawing ability of the substituent increases. For this reason, it is not surprising that a very good linear relationship (correlation coefficient, R^2^ = 0.93) was found when plotting the computed ΔE_int_ values versus the corresponding σ_p_-Hammett substituent constants [31], which are a measure of the relative electron donor/withdrawing ability of a substituent (see Figure 4). Similarly, the electrostatic attractions, ΔV_elstat_, follows a similar trend as ΔE_int_ and the **4-NO_2_·Cl^−^** system exhibits the strongest electrostatic attraction (ca. 12 kcal/mol higher than that in the parent **4·Cl^−^**), whereas the complex having the donor NMe_2_ substituent has the lowest (i.e., less negative) ΔV_elstat_ value. As expected, a similar linear correlation as ΔE_int_ was found for this energy contributor (R^2^ = 0.94, Figure 4).

**Figure 5:**
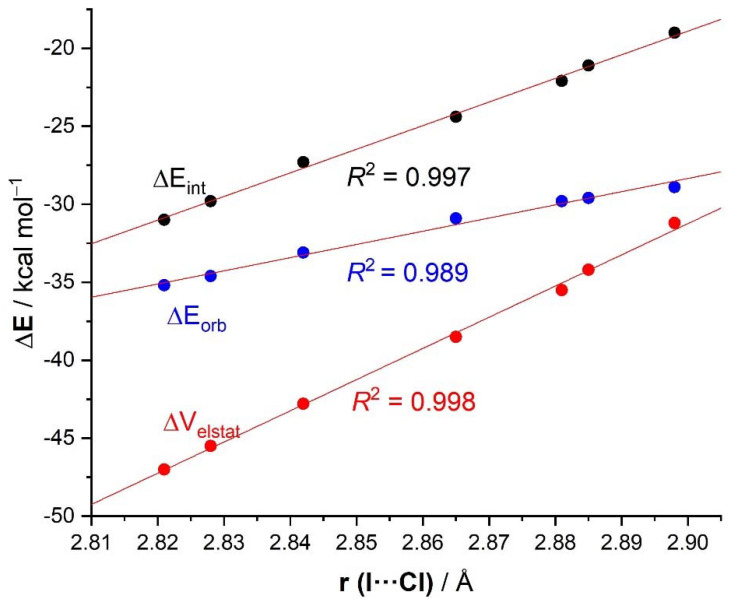
Plot of the energy terms, ΔE_int_ (black circles), ΔV_elstat_ (red circles), and ΔE_orb_ (blue circles) vs the computed I⋯Cl bond lengths.

Although a similar trend was also found for the ΔE_orb_ term, the correlation involving the ΔE_orb_ values is not only slightly worse (R^2^ = 0.87) but more importantly, its slope is clearly lower than those computed for the ΔE_int_ or ΔV_elestat_ terms (Figure 4). This suggests that the impact of the nature of the substituents on the orbital attractions is comparatively less significant than on the electrostatic interactions, the latter being the main contributor to the total interaction energy in all cases (52–57% to ΔE_int_, see Table 2). Despite that, the EDA-NOCV method (as well as the NBO-SOPT approach) clearly shows that the dominant LP(Cl^−^)→σ*(I—C) molecular orbital interaction also follows the same trend, which indicates that the nature of the *para*-substituent not only modifies the σ-hole of the iodine atom but also the acceptor capacity of the key σ*(I—C) molecular orbital. Finally, we found once again that the strength of these hydrogen bond-enhanced halogen bonds is nicely reflected in the computed I⋯Cl bond lengths. Thus, the shortest distance was computed for the **4-NO_2_·Cl^−^** system, which exhibits the highest ΔE_int_ value, whereas the **4-NMe_2_·Cl^−^** complex, where the interaction is lowest, exhibits the longest bond length. As a result, excellent linear correlations were found when plotting this geometrical parameter versus the computed ΔE_int_ as well as its main contributors (Figure 5). The correlation involving ΔE_orb_ has once again a lower slope, which is consistent with the above results.

For completeness, we also included in Table 2 the experimentally prepared trifluorinated and pyridinium systems **1** and **6,** where the bulky CPh_3_ group was replaced by a CH_3_ group [14]. According to the EDA data, the effect of the three fluorine atoms in the aryl group of **1** is comparable to that of the *p*-NO_2_ group, as the computed ΔE_int_, as well as its main energy contributors, are rather similar (see Table 2). At variance, the **6·Cl^−^** complex, featuring a positively charged pyridinium moiety, exhibits a remarkably strong halogen bond, which is ca. three times as strong as the **4-NO_2_·Cl^−^** system (ΔΔE_int_ = 74.1 kcal/mol with respect to the parent **4·Cl^−^**). This is not surprising if we take into account the highly electron-withdrawing nature of the cationic pyridinium group, which significantly enhances the electrophilicity of the iodine atom. This markedly strong interaction is the result of a significant increase of both the electrostatic and orbital (albeit again to a lesser extent) interactions (ΔΔV_elstat_ = 85.5 kcal/mol and ΔΔE_orb_ = 31.0 kcal/mol, with respect to the parent **4·Cl^−^**). The computed remarkably high interaction in **6·Cl^−^** is again reflected in a markedly short I···Cl distance of only 2.632 Å, which is significantly shorter than that of the parent **4·Cl^−^** system (2.881 Å).

To complete this study, we were curious to analyze the effect of replacing the amide group, responsible for the intramolecular hydrogen bond, by a thioamide group on the relative strength of the enhanced halogen bond. This was motivated by the well-known superior activity of related thioureas over their urea counterpart in organocatalysis [32,33,34], in line with their comparatively stronger acidity (for instance, pK_a_ = 21.1 and 26.9, for thiourea and urea, respectively) [35]. Therefore, with the help of the EDA-NOCV method, we analyzed the nature of the halogen bond involving the thioamide analogues of the parent **4·Cl^−^** and **5·Cl^−^** systems (Table 3).

From the data in Table 3, it becomes clear that the replacement of the amide group in **4·Cl^−^** by a thioamide group (**7·Cl^−^**) further enhances the I···Cl halogen bond strength. Thus, the computed interaction energy becomes ca. 3 kcal/mol higher than that computed for the parent amide system (**4·Cl^−^**) and ca. 6 kcal/mol higher than its non-hydrogen bond counterpart **3·Cl^−^** complex. This is again the result of an enhancement of the electrostatic and orbital (mainly the key LP(Cl^−^)→σ*(I—C) interaction) attractions. In line with the above results, the presence of an additional thioamide hydrogen donor (complex **8·Cl^−^**) leads to an even more pronounced enhancement of the halogen bond strength as confirmed by the computed high ΔE_int_ of −35.5 kcal/mol (that is ca. 17 kcal/mol higher than the interaction in **3·Cl^−^**), which is even stronger than the interaction in the system having the highly electron-withdrawing NO_2_ group **4-NO_2_·Cl^−^**. Once again, the relative halogen bond strength is reflected in the computed I···Cl distances, which are shorter for the thioamide systems as compared to their amide analogues: 2.881 Å (**4·Cl^−^**) > 2.855 Å (**7·Cl^−^**) > 2.838 Å (**5·Cl^−^**) > 2.796 Å (**8·Cl^−^**). For completeness, we also considered the analogous selenoamide **9·Cl^−^**. Following the expected trend, the computed interaction is even stronger than that in the corresponding thioamide, which is also reflected in a shorter I···Cl distance of 2.847 Å.

## 3. Computational Methods

Geometry optimization of all complexes was performed without symmetry constraints using the Gaussian09 [36] suite of programs at the B3LYP [37,38,39] /def2-TZVPP [40] level of theory using the D3 dispersion correction suggested by Grimme et al. [41]. This level is denoted as B3LYP-D3/def2-TZVPP. All species were also characterized by frequency calculations and have positive definite Hessian matrices, thus confirming that the computed structures are minima on the potential energy surface. Wiberg Bond Indices (WBIs) have been computed using the natural bond orbital (NBO) method [15]. All QTAIM [16] results described in this work correspond to calculations performed at the B3LYP-D3/def2-TZVPP/WTBS [42] (for I) level on the optimized geometry obtained at the B3LYP-D2/def2-TZVPP level. The topology of the electron density was conducted using the AIMAll program package [43]. Cartesian coordinates and total energies of all species described in the manuscript are available in the Supplementary Materials.

The interaction between the selected fragments has been investigated with the EDA-NOCV method, which combines the energy decomposition analysis (EDA) [18,19] with the natural orbitals for chemical valence (NOCV) [28] methods. Within this approach, the interaction energy can be decomposed into the following physically meaningful terms:

ΔE_int_ = ΔV_elstat_ + ΔE_Pauli_ + ΔE_orb_+ ∆E_disp_ (1)

The term ΔV_elstat_ corresponds to the classical electrostatic interaction between the unperturbed charge distributions of the deformed reactants and is usually attractive. The Pauli repulsion ΔE_Pauli_ comprises the destabilizing interactions between occupied orbitals and is responsible for any steric repulsion. The orbital interaction ΔE_orb_ accounts for charge transfer (interaction between occupied orbitals on one moiety with unoccupied orbitals on the other, including HOMO–LUMO interactions) and polarization (empty-occupied orbital mixing on one fragment due to the presence of another fragment). Finally, the ∆E_disp_ term takes into account the interactions, which are due to dispersion forces. Note that the concepts of Pauli repulsion and orbital interaction that feature in our canonical EDA have been also successfully applied to systems that were studied using other decomposition schemes such as DFT-SAPT [22] or valence bond (VB) theory [44,45].

The EDA-NOCV method makes it possible to further partition the total orbital interactions into pairwise contributions of the orbital interactions. Details of the method can be found in the literature [18,19].

The EDA-NOCV calculations were carried out using the B3LYP-D3/def2-TZVPP optimized geometry with the program package ADF 2020.01 [46,47] using the same functional (B3LYP-D3) in conjunction with a triple-ζ-quality basis set using uncontracted Slater-type orbitals (STOs) augmented by two sets of polarization function with a frozen-core approximation for the core electrons [48]. An auxiliary set of s, p, d, f, and g STOs were used to fit the molecular densities and to represent the Coulomb and exchange potentials accurately in each SCF cycle [49]. Scalar relativistic effects were incorporated by applying the zeroth-order regular approximation (ZORA) [50,51,52]. This level of theory is denoted as ZORA-B3LYP-D3/TZ2P//B3LYP-D3/def2-SVP.

## 4. Conclusions

From the computational study reported herein, the following conclusions can be drawn: (1) The strength of a halogen bond is enhanced by ca. 3 kcal/mol when the halogen donor simultaneously operates as a halogen bond donor and a hydrogen bond acceptor. (2) The presence of a second hydrogen bond further increases the halogen bond strength but not in an additive manner. (3) This enhancement is the result of both stronger electrostatic and orbital (mainly the LP(Cl^-^)→σ*(I—C) molecular orbital interaction) attractions between the XB donor and the XB acceptor. (4) The halogen bond strength can be easily tuned by modifying the electron density of the aryl group of the XB donor. Thus, electron-withdrawing groups, such as a NO_2_ or pyridinium groups, significantly increase the electrophilicity of the system and lead to rather strong halogen bonds. (5) The strength of the halogen bond can be also further enhanced by replacing the amide group responsible for the hydrogen bond by a more acidic thioamide or selenoamide group. (6) In all cases, the relative strength of the HBeXB is nicely reflected in the corresponding I···Cl bond lengths, which become shorter and shorter as the HBeXB strength increases.

## Figures and Tables

**Table 1:** EDA results (in kcal/mol) computed at the ZORA-B3LYP-D3/TZ2P//B3LYP-D3/def2-SVP level.

Complex	3·Cl^−^	4·Cl^−^	5·Cl^−^
ΔE_int_	−19.0	−22.1	−28.8
ΔE_Pauli_	42.0	43.6	49.7
ΔV_elstat_^a^	−31.8 (52.2%)	−35.5 (54.0%)	−45.1 (57.4%)
ΔE_orb_^a^	−28.7 (47.0%)	−29.8 (45.4%)	−33.0 (42.0%)
ΔE_orb(1)_^b^	−20.6 (71.8%)	−21.4 (71.8%)	−24.6 (74.5%)
ΔE_orb(2)_^b^	−2.4 (8.4%)	−2.3 (7.7%)	−2.3 (7.0%)
ΔE_orb(rest)_^b^	−5.8 (20.2%)	−6.1 (20.5%)	−8.4 (25.5%)
ΔE_disp_^a^	−0.5 (0.8%)	−0.4 (0.6%)	−0.5 (0.6%)

^a^ The values within parentheses indicate the percentage to the total attractive interactions, ΔE_int_ = ΔV_elstat_ + ΔE_orb_ +ΔE_disp_. ^b^ The values within parentheses indicate the percentage to the total orbital interactions (ΔE_orb_).

**Table 2:** EDA results (in kcal/mol) computed at the ZORA-B3LYP-D3/TZ2P//B3LYP-D3/def2-SVP level.

Complex	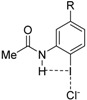							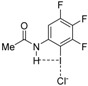	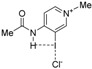
	4·Cl^−^	4−NMe_2_·Cl^−^	4−OMe·Cl^−^	4−F·Cl^−^	4−CF_3_·Cl^−^	4−CN·Cl^−^	4−NO_2_·Cl^−^	1·Cl^−^	6·Cl^−^
ΔE_int_	−22.1	−19.0	−21.1	−24.4	−27.3	−29.8	−31.0	−31.1	−96.2
ΔE_Pauli_	43.6	41.5	43.1	45.4	48.6	50.6	51.6	54.5	86.0
ΔV_elstat_^a^	−35.5 (54.0%)	−31.2 (51.6%)	−34.2 (53.3%)	−38.5 (55.2%)	−42.8 (56.1%)	−45.5 (56.5%)	−47.0 (56.9%)	−48.1 (56.2%)	−121.0 (66.4%)
ΔE_orb_^a^	−29.8 (45.4%)	−28.9 (47.8%)	−29.6 (46.1%)	−30.9 (44.3%)	−33.1 (43.4%)	−34.6 (43.0%)	−35.2 (42.6%)	−37.1 (43.3%)	−60.8 (33.4%)
ΔE_orb(1)_^b^	−21.4 (71.8%)	−20.2 (69.9%)	−21.1 (71.3%)	−22.3 (72.2%)	−23.8 (71.9%)	−24.7 (71.4%)	−25.0 (71.0%)	−27.5 (74.1%)	−46.2 (76.0%)
ΔE_orb(2)_^b^	−2.3 (7.7%)	−2.5 (8.7%)	−2.3 (7.8%)	−2.3 (7.4%)	−2.6 (7.9%)	−3.0 (8.7%)	−3.4 (9.7%)	−2.4 (6.5%)	−3.6 (5.9%)
ΔE_orb(rest)_^b^	−6.1 (20.5%)	−6.1 (21.1%)	−6.2 (20.9%)	−6.3 (20.4%)	−6.6 (19.9%)	−6.8 (19.7%)	−6.8 (19.3%)	−7.2 (19.4%)	−11.0 (18.1%)
ΔE_disp_	−0.4 (0.6%)	−0.4 (0.7%)	−0.4 (0.6%)	−0.4 (0.6%)	−0.4 (0.5%)	−0.4 (0.5%)	−0.4 (0.5%)	−0.4 (0.5%)	−0.4 (0.2%)
r(I···Cl)/Å	2.881	2.898	2.885	2.865	2.842	2.828	2.821	2.796	2.632

^a^ The values within parentheses indicate the percentage to the total attractive interactions, ΔE_int_ = ΔV_elstat_ + ΔE_orb_ +ΔE_disp_. ^b^ The values within parentheses indicate the percentage to the total orbital interactions (ΔE_orb_).

**Table 3:** EDA results (in kcal/mol) computed at the ZORA-B3LYP-D3/TZ2P//B3LYP-D3/def2-SVP level.

	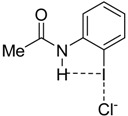	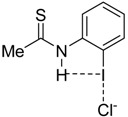	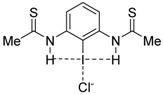	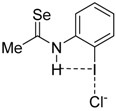
	4·Cl^−^	7·Cl^−^	8·Cl^−^	9·Cl^−^
ΔEint	−22.1	−24.9	−35.5	−25.8
ΔEPauli	43.6	47.0	56.2	48.2
ΔVelstat ^a^	−35.5 (54.0%)	−39.2 (54.5%)	−52.0 (56.8%)	−40.4 (54.7%)
ΔE_orb_^a^	−29.8 (45.4%)	−32.3 (44.9%)	−39.0 (42.6%)	−33.1 (44.8%)
ΔE_orb(1)_^b^	−21.4 (71.8%)	−23.1 (71.5%)	−27.8 (71.3%)	−23.6 (71.2%)
ΔE_orb(2)_^b^	−2.3 (7.7%)	−2.4 (7.4%)	−2.5 (6.4%)	−2.4 (7.3%)
ΔE_orb(rest)_^b^	−6.1 (20.5%)	−6.8 (21.1%)	−8.7 (22.3%)	−7.1 (21.5%)
ΔE_disp_	−0.4 (0.6%)	−0.4 (0.6%)	−0.5 (0.5%)	−0.4 (0.5%)
r(I···Cl)/Å	2.881	2.855	2.796	2.847

^a^ The values within parentheses indicate the percentage to the total attractive interactions, ΔE_int_ = ΔV_elstat_ + ΔE_orb_ +ΔE_disp_. ^b^ The values within parentheses indicate the percentage to the total orbital interactions (ΔE_orb_).

## Data Availability

Not applicable.

## Supplementary Materials

The following are available online, Cartesian coordinates and total energies of all species described in the manuscript.
