# Supplementary material for: Nature of the Hydrogen Bond Enhanced Halogen Bond
Source: Molecules. 2021 Mar 26;26(7):1885. doi: 10.3390/molecules26071885 (PMC8036253; doi:10.3390/molecules26071885)
Supplement: Supplementary file 1 [file molecules-26-01885-s001.pdf]

Nature of the Hydrogen Bond Enhanced Halogen Bond

Susana Portela and Israel Fernández*

S. Portela and I. Fernández

Departamento de Química Orgánica I and Centro de Innovación en Química Avanzada (ORFEO-CINQA)

Facultad de Ciencias Químicas

Universidad Complutense de Madrid, 28040-Madrid, Spain

e-mail: israel@quim.ucm.es

Contents:

1. Cartesian coordinates and energies…………………………………………………....

Cartesian coordinates (in Å) and total energies (in a.u., noncorrected ZVPE included) of all the stationary points discussed in the text. All calculations have been performed at the B3LYP-D3/def2-TZVPP level.

1·Cl$^-$**:** E= -1495.702740

I	1.739697000	-0.501487000	0.000020000
O	-2.049783000	3.599183000	-0.000034000
C	-2.598476000	-1.660966000	-0.000010000
N	-0.466562000	1.939053000	0.000093000
H	0.533314000	1.779440000	0.000048000
C	-1.208045000	-1.636901000	0.000034000
C	-0.482915000	-0.461799000	0.000055000
C	-1.210504000	0.746752000	0.000041000
C	-2.608734000	0.751196000	-0.000004000
H	-3.161522000	1.675609000	-0.000021000
C	-3.277500000	-0.456511000	-0.000035000
F	-0.585164000	-2.832679000	0.000047000
F	-4.628820000	-0.474778000	-0.000083000
F	-3.270839000	-2.828811000	-0.000027000
C	-0.882718000	3.236767000	0.000012000
Cl	4.532917000	-0.365741000	-0.000083000
C	0.247882000	4.251517000	-0.000005000
H	1.242588000	3.808184000	0.000107000
H	0.137954000	4.886131000	-0.879225000
H	0.137825000	4.886287000	0.879087000

3·Cl$^-$**:** E= -1217.713877

I	1.140307000	-0.579082000	-0.014347000
O	-1.441496000	2.312966000	1.341349000
C	-3.012452000	-2.202563000	0.227576000
O	-1.309727000	1.456754000	-0.750892000
C	-1.629531000	-2.049725000	0.250960000
C	-1.027055000	-0.826427000	-0.048115000
C	-1.872515000	0.232287000	-0.372307000
C	-3.252469000	0.102620000	-0.399560000
H	-3.856072000	0.964308000	-0.652434000
C	-3.830175000	-1.125026000	-0.098328000
H	-1.000240000	-2.893433000	0.505219000
H	-4.906885000	-1.235493000	-0.114572000
H	-3.451043000	-3.164700000	0.463721000
C	-1.000337000	2.356552000	0.226091000
Cl	4.001927000	-0.182627000	0.011350000
C	-0.028178000	3.373148000	-0.299238000
H	0.954765000	2.898113000	-0.332788000
H	-0.286829000	3.672712000	-1.313653000
H	0.003338000	4.231524000	0.365806000

4·Cl$^-$**:** E= -1197.840262

I	-1.452402000	-0.316935000	0.000151000
O	3.572279000	2.111516000	-0.000092000
C	2.098876000	-3.003733000	-0.000023000
N	1.486934000	1.150309000	-0.000024000
H	0.500761000	1.378180000	0.000025000
C	0.815193000	-2.464884000	0.000041000
C	0.604991000	-1.090288000	0.000043000
C	1.727245000	-0.237367000	-0.000024000
C	3.019380000	-0.774840000	-0.000090000
H	3.864513000	-0.106308000	-0.000141000
C	3.196778000	-2.152917000	-0.000089000
H	-0.042969000	-3.125218000	0.000092000

H	4.201850000	-2.556079000	-0.000140000
H	2.235308000	-4.078362000	-0.000023000
C	2.352654000	2.200534000	-0.000052000
Cl	-4.137157000	0.727341000	-0.000341000
C	1.676292000	3.562496000	0.000001000
H	0.588031000	3.518795000	-0.000005000
H	2.013112000	4.111681000	0.879201000
H	2.013122000	4.111752000	-0.879150000

4-F·Cl$^-$: E= -1297.133254

I	-1.647067000	-0.440118000	0.000115000
O	2.938363000	2.751068000	-0.000162000
C	2.295655000	-2.524742000	0.000032000
N	1.026212000	1.482245000	-0.000051000
H	0.016110000	1.550429000	-0.000022000
C	0.942804000	-2.189648000	0.000092000
C	0.511431000	-0.869040000	0.000057000
C	1.482502000	0.153444000	-0.000033000
C	2.846432000	-0.161474000	-0.000097000
H	3.592957000	0.614780000	-0.000168000
C	3.211697000	-1.492579000	-0.000065000
H	0.205427000	-2.981873000	0.000172000
H	2.631734000	-3.552335000	0.000060000
C	1.720177000	2.655348000	-0.000111000
Cl	-4.457892000	0.116458000	-0.000095000
C	0.843094000	3.896418000	-0.000109000
H	-0.225375000	3.685756000	-0.000105000
H	1.091570000	4.490706000	0.879089000
H	1.091566000	4.490705000	-0.879308000
F	4.540176000	-1.793592000	-0.000128000

4-CN·Cl$^-$: E= -1290.130399

I	-1.786712000	-0.542527000	-0.000117000
O	2.484257000	3.049994000	0.000086000
C	2.321478000	-2.262655000	0.000082000
N	0.697205000	1.612722000	-0.000055000
H	-0.314591000	1.587700000	-0.000106000
C	0.950464000	-2.054712000	0.000052000
C	0.402372000	-0.775303000	-0.000009000
C	1.277097000	0.333381000	-0.000020000
C	2.658996000	0.141572000	-0.000003000
H	3.316985000	0.994442000	-0.000024000
C	3.177119000	-1.157484000	0.000046000
H	0.285243000	-2.908562000	0.000087000
H	2.730010000	-3.263985000	0.000132000
C	1.280628000	2.846413000	-0.000047000
Cl	-4.596796000	-0.225899000	0.000244000
C	0.293160000	4.000832000	0.000022000
H	-0.751258000	3.692097000	-0.000055000
H	0.485918000	4.615195000	0.879249000
H	0.486003000	4.615357000	-0.879071000
C	4.592007000	-1.355534000	0.000058000
N	5.732650000	-1.532296000	0.000068000

4-NO_2·Cl$^-$: E= -1402.441193

I	-2.041275000	-0.598171000	0.000000000
O	1.978164000	3.272161000	-0.000017000
C	2.169819000	-2.042744000	-0.000010000
N	0.290652000	1.719693000	0.000044000
H	-0.717048000	1.627872000	0.000022000
C	0.788456000	-1.924553000	-0.000012000

C	0.155760000	-0.683340000	-0.000005000
C	0.955023000	0.482861000	0.000017000
C	2.346001000	0.384539000	0.000016000
H	2.958597000	1.268900000	0.000029000
C	2.926456000	-0.878244000	0.000001000
H	0.181930000	-2.820788000	-0.000013000
H	2.657849000	-3.004696000	-0.000016000
C	0.791417000	2.990321000	0.000071000
Cl	-4.858874000	-0.461415000	-0.000002000
C	-0.270985000	4.076316000	-0.000049000
H	-1.292673000	3.699091000	-0.000100000
H	-0.119498000	4.702279000	0.879054000
H	-0.119386000	4.702212000	-0.879182000
N	4.388882000	-0.980983000	-0.000001000
O	4.888992000	-2.103042000	-0.000014000
O	5.046624000	0.052174000	0.000000000

4-OMe·Cl$^-$: E= -1312.381320

I	1.828565000	-0.627444000	-0.000011000
O	-2.078771000	3.363960000	0.000015000
C	-2.435023000	-1.944341000	0.000000000
N	-0.443643000	1.752432000	0.000027000
H	0.561226000	1.630230000	-0.000007000
C	-1.040682000	-1.863532000	-0.000003000
C	-0.368403000	-0.653092000	-0.000002000
C	-1.139788000	0.530358000	0.000010000
C	-2.531983000	0.469953000	0.000010000
H	-3.117876000	1.373820000	0.000020000
C	-3.173416000	-0.766428000	0.000004000
H	-0.467245000	-2.781969000	0.000001000
H	-2.910739000	-2.913720000	0.000005000
C	-0.901838000	3.035157000	0.000055000
Cl	4.712739000	-0.571489000	0.000024000
C	0.196810000	4.086405000	-0.000050000
H	1.205597000	3.675933000	-0.000030000
H	0.066302000	4.717181000	-0.879297000
H	0.066319000	4.717325000	0.879096000
O	-4.547308000	-0.712066000	0.000009000
C	-5.259432000	-1.929158000	-0.000011000
H	-5.041081000	-2.529323000	-0.890103000
H	-5.041107000	-2.529336000	0.890078000
H	-6.315269000	-1.664401000	-0.000025000

4-NMe$_2$·Cl$^-$: E= -1331.791777

I	2.110233000	-0.613299000	0.003801000
O	-1.873237000	3.305984000	0.039120000
C	-2.130420000	-1.993571000	0.100077000
N	-0.207052000	1.724427000	0.016413000
H	0.800010000	1.623278000	-0.005168000
C	-0.743163000	-1.896090000	0.078148000
C	-0.081069000	-0.677257000	0.055511000
C	-0.874895000	0.484144000	0.050973000
C	-2.268075000	0.405493000	0.071378000
H	-2.816484000	1.328864000	0.058819000
C	-2.921009000	-0.834712000	0.110582000
H	-0.159958000	-2.808645000	0.072558000
H	-2.577140000	-2.976160000	0.109790000
C	-0.689251000	2.996033000	0.014266000
Cl	5.005059000	-0.492976000	-0.075266000
C	0.387762000	4.068727000	-0.022796000
H	1.404027000	3.677780000	-0.045963000

H	0.222117000	4.689821000	-0.903041000
H	0.268115000	4.704164000	0.854726000
N	-4.322590000	-0.900181000	0.176554000
C	-4.955086000	-2.158573000	-0.158360000
H	-6.033899000	-2.054731000	-0.053401000
H	-4.737176000	-2.492636000	-1.184413000
H	-4.637188000	-2.948568000	0.522974000
C	-5.075702000	0.289182000	-0.173252000
H	-4.905898000	0.616944000	-1.209375000
H	-6.138110000	0.087128000	-0.044374000
H	-4.817941000	1.120374000	0.482041000

4-CF$_3$·Cl$^-$: E= -1535.040986

I	2.309154000	-0.635897000	-0.000189000
O	-1.556404000	3.392471000	0.000771000
C	-1.962800000	-1.902162000	-0.027341000
N	0.067640000	1.772515000	-0.012423000
H	1.071130000	1.640686000	-0.010948000
C	-0.573964000	-1.845053000	-0.017691000
C	0.107863000	-0.633881000	-0.011533000
C	-0.643961000	0.560631000	-0.016241000
C	-2.039354000	0.514797000	-0.027652000
H	-2.599933000	1.434768000	-0.036803000
C	-2.687138000	-0.716816000	-0.035458000
H	-0.006181000	-2.766545000	-0.016684000
H	-2.473271000	-2.854623000	-0.035862000
C	-0.381250000	3.060299000	-0.002370000
Cl	5.150789000	-0.617996000	0.014404000
C	0.723211000	4.103766000	0.004959000
H	1.729196000	3.686668000	0.000989000
H	0.595964000	4.742343000	-0.868989000
H	0.597250000	4.728224000	0.889236000
C	-4.183956000	-0.743949000	0.003765000
F	-4.746081000	0.218831000	-0.762425000
F	-4.696292000	-1.925116000	-0.419081000
F	-4.672839000	-0.553296000	1.259546000

4-4F·Cl$^-$: E= -1594.977160

I	-1.870709000	-0.498270000	0.028369000
O	1.531757000	3.106080000	-1.110292000
C	2.437720000	-1.772989000	-0.109822000
N	0.421587000	1.860040000	0.454796000
H	-0.507433000	1.753548000	0.840203000
C	1.050345000	-1.724274000	-0.129884000
C	0.353388000	-0.543185000	0.041685000
C	1.090010000	0.633367000	0.256236000
C	2.479704000	0.584787000	0.321732000
C	3.153974000	-0.610290000	0.120286000
C	0.620439000	2.983283000	-0.318161000
Cl	-4.643686000	-0.276995000	0.073989000
C	-0.417264000	4.064479000	-0.076159000
H	-1.415123000	3.692263000	-0.317083000
H	-0.419890000	4.370595000	0.971103000
H	-0.186131000	4.918457000	-0.705798000
F	4.496144000	-0.648271000	0.175704000
F	3.203970000	1.671373000	0.631486000
F	3.096119000	-2.931280000	-0.298523000
F	0.404185000	-2.889336000	-0.340500000

5·Cl$^-$: E= -1405.906417

I	-0.000380000	1.451849000	-0.000054000

O	-4.084826000	-2.320589000	0.002558000
C	1.211057000	-2.890610000	-0.000004000
N	-2.407352000	-0.749362000	-0.001512000
H	-2.257947000	0.249826000	-0.002482000
C	1.209809000	-1.491502000	0.000057000
C	0.000256000	-0.775434000	-0.000275000
C	-1.208965000	-1.492100000	-0.000836000
C	-1.209481000	-2.891205000	-0.001012000
H	-2.147527000	-3.419829000	-0.001407000
C	0.000957000	-3.566592000	-0.000525000
H	0.001208000	-4.649302000	-0.000602000
H	2.149400000	-3.418728000	0.000371000
C	-3.707069000	-1.158700000	0.000508000
Cl	-0.001434000	4.290080000	0.000215000
C	-4.714214000	-0.018882000	-0.000272000
H	-4.265634000	0.973664000	-0.000386000
H	-5.349379000	-0.124721000	-0.879652000
H	-5.350133000	-0.124279000	0.878589000
N	2.407864000	-0.748177000	0.000369000
H	2.257947000	0.250933000	0.000387000
C	3.707717000	-1.156876000	0.000367000
O	4.086068000	-2.318614000	0.000393000
C	4.714466000	-0.016721000	0.000108000
H	4.265723000	0.975748000	0.000126000
H	5.350250000	-0.122206000	0.879068000
H	5.349877000	-0.122309000	-0.879112000

6·Cl⁻: E= -1253.678042

I	-1.615356000	-0.403857000	0.000021000
O	2.866514000	3.034949000	-0.000138000
N	2.521490000	-2.166423000	0.000037000
N	1.016550000	1.683390000	-0.000030000
H	0.000663000	1.692172000	-0.000003000
C	1.179377000	-1.929255000	0.000070000
C	0.643115000	-0.670711000	0.000060000
C	1.552241000	0.420398000	0.000011000
C	2.936185000	0.168393000	-0.000062000
H	3.644829000	0.978975000	-0.000151000
C	3.381702000	-1.128991000	-0.000014000
H	0.555607000	-2.813064000	0.000130000
H	4.433278000	-1.370424000	-0.000081000
C	1.660896000	2.918290000	-0.000039000
Cl	-4.226424000	-0.074067000	-0.000082000
C	0.703673000	4.085460000	0.000134000
H	0.059064000	4.051801000	0.880236000
H	1.274352000	5.008438000	-0.000038000
H	0.058502000	4.051740000	-0.879540000
C	3.003794000	-3.553545000	-0.000019000
H	2.641364000	-4.067537000	0.888092000
H	2.641731000	-4.067368000	-0.888382000
H	4.089403000	-3.559762000	0.000203000

7·Cl⁻: E= -1520.790787

I	1.708328000	0.191919000	0.000044000
S	-3.992488000	-1.657376000	-0.000083000
C	-1.387847000	3.393252000	-0.000073000
N	-1.415607000	-0.805379000	0.000036000
H	-0.450591000	-1.125069000	0.000045000
C	-0.205505000	2.658355000	-0.000044000
C	-0.215552000	1.268488000	-0.000010000
C	-1.461506000	0.605151000	-0.000004000

C	-2.650657000	1.340192000	-0.000034000
H	-3.591728000	0.815834000	-0.000031000
C	-2.607018000	2.728644000	-0.000069000
H	0.745931000	3.174835000	-0.000046000
H	-3.535309000	3.285855000	-0.000092000
H	-1.351864000	4.475944000	-0.000100000
C	-2.329465000	-1.787058000	0.000049000
Cl	4.203582000	-1.194682000	0.000033000
C	-1.699556000	-3.167089000	-0.000044000
H	-0.608289000	-3.140420000	-0.000081000
H	-2.039346000	-3.716557000	0.877018000
H	-2.039408000	-3.716479000	-0.877132000

8·Cl$^-$: E= -2051.806042

I	-0.000066000	1.656911000	-0.000052000
S	4.461001000	-2.305358000	-0.051191000
C	-1.208827000	-2.703963000	-0.010942000
N	2.396935000	-0.540850000	0.024969000
H	2.184477000	0.450446000	0.048296000
C	-1.209412000	-1.306199000	-0.009946000
C	0.000077000	-0.586939000	0.000221000
C	1.209633000	-1.306082000	0.010593000
C	1.209164000	-2.703847000	0.011979000
H	2.146808000	-3.232973000	0.020037000
C	0.000199000	-3.380178000	0.000608000
H	0.000250000	-4.462422000	0.000755000
H	-2.146429000	-3.233166000	-0.018887000
C	3.711228000	-0.819007000	0.004969000
Cl	-0.000471000	4.452469000	-0.000310000
C	4.565269000	0.433951000	0.060397000
H	3.986944000	1.353100000	-0.053141000
H	5.085188000	0.467182000	1.018281000
H	5.323554000	0.390961000	-0.718629000
N	-2.396779000	-0.541065000	-0.024380000
H	-2.184399000	0.450257000	-0.047195000
C	-3.711038000	-0.819368000	-0.005177000
S	-4.460703000	-2.305840000	0.049447000
C	-4.565331000	0.433506000	-0.058992000
H	-3.986367000	1.353020000	0.048104000
H	-5.091755000	0.463751000	-1.013355000
H	-5.318407000	0.392896000	0.725278000

9·Cl$^-$: E= -3524.156130

I	-2.197757000	0.051923000	-0.003660000
Se	3.858476000	-0.945777000	-0.025737000
C	0.380479000	3.687035000	0.000743000
N	1.033756000	-0.459273000	0.028122000
H	0.120059000	-0.909777000	0.042114000
C	-0.679035000	2.784071000	-0.004364000
C	-0.460428000	1.411417000	0.003726000
C	0.871407000	0.944169000	0.017292000
C	1.937394000	1.847278000	0.023739000
H	2.947552000	1.471343000	0.034287000
C	1.685843000	3.213264000	0.015145000
H	-1.697043000	3.152375000	-0.014965000
H	2.519876000	3.903449000	0.019914000
H	0.183203000	4.752192000	-0.006218000
C	2.062585000	-1.306658000	0.016213000
Cl	-4.422045000	-1.724507000	-0.012179000
C	1.626859000	-2.755509000	0.057494000
H	0.545151000	-2.874344000	-0.038928000

H	2.119076000	-3.310347000	-0.738796000
H	1.942900000	-3.199292000	1.001900000
